# Effect of antipyretics on performance of influenza surveillance in Ghana

**DOI:** 10.1111/irv.13068

**Published:** 2022-11-12

**Authors:** Joseph Asamoah Frimpong, William Kwabena Ampofo, Kofi Mensah Nyarko, Jazmin Duque, James Aboagye, Kwadwo Koram, Marc‐Alain Widdowson

**Affiliations:** ^1^ Ghana Field Epidemiology and Laboratory Training Program University of Ghana Accra Ghana; ^2^ Noguchi Memorial Institute for Medical Research University of Ghana Accra Ghana; ^3^ Department of Environmental and Public Health University of Environmental and Sustainable Development Somanya Ghana; ^4^ Abt Associates Rockville Maryland USA; ^5^ Institute of Tropical Medicine Antwerp Belgium

**Keywords:** case definition, fever, influenza

## Abstract

**Background:**

The World Health Organization's case definition for influenza‐like illness (ILI) includes a measured temperature of ≥38°C. We conducted this study to assess the effect of antipyretics on performance of ILI surveillance in Ghana.

**Methods:**

A cross‐sectional study was conducted in two districts of Ghana from September 2013 to May 2014. We collected epidemiological data and respiratory specimens from an expanded ILI case definition, which included patients presenting to health facilities with measured temperature ≥38°C or reported fever (but afebrile at the time of evaluation), and cough, with onset in the last 10 days. Specimens were tested for influenza viruses by real time reverse‐transcription polymerase chain reaction.

**Results:**

Of 321 participants who met our expanded ILI case definition, 236 presented with temperature of <38°C but reported subjective fever. Of these, 17% (39/236) were positive for influenza virus; Of those with fever ≤38°C who took antipyretics, 21%(16/77) were positive for influenza, compared with 14%(23/159) of those who did not take antipyretics. The addition of subjective fever to the standard ILI case definition captured approximately an additional 57% influenza cases but also required testing of approximately four times as many patients. However, including those without fever on presentation that had taken antipyretics found an additional 23% of Influenza cases and only two times as much testing.

**Conclusion:**

Depending on the goals of surveillance (monitoring virus circulation or determining disease burden) and available resources, a more sensitive case definition including subjective fever and history of use of antipyretics may be warranted.

## 1 INTRODUCTION

An estimated 20% of children and 5% of adults experience influenza infection each year.^1^, ^2^ Influenza surveillance systems are important for several reasons, including: (1) to understand circulating seasonal strains for vaccine policy decisions, (2) to determine the preventable burden of influenza, and (3) to detect novel variants of influenza viruses.^3^ Surveillance in Ghana has shown that influenza viruses are identifiable year round with biannual epidemics occurring during the rainy seasons (May to October).^4^ Although influenza has been long overlooked in Africa as a cause of human illness, improved surveillance in recent years indicates that it causes substantial morbidity and mortality on the continent, particularly among certain high‐risk groups.^5^

Determining the best case definition for influenza surveillance systems will depend on the objective but remains a challenge since symptoms of influenza virus infection are non‐specific (e.g., fever, cough, and body aches) and infection must be confirmed using molecular laboratory methods, such as reverse transcription polymerase chain reaction (RT‐PCR), or viral culture.^6^, ^7^, ^8^ For characterization of circulating strains, or understanding circulation and seasonality, a specific case definition is most efficient since only a representative and systematic selection of cases and strains is required. But for estimation of burden, the optimal case definition needs to be both sensitive to capture most influenza cases but reasonably specific, in order to reduce cost of surveillance and of laboratory testing.^9^, ^10^ Also important is to understand the factors that may lead to an underestimate of burden assessed through surveillance. The World Health Organization (WHO) standard case definition for influenza‐like illness (ILI) is a patient with measured fever ≥38°C, and cough, with illness onset in the last 10 days. This case definition aims to provide a balance between sensitivity and specficity^11^ but was largely designed for strain surveillance. For severe acute respiratory illness (SARI) surveillance, the WHO standard case definition is a patient hospitalized with history of fever or measured fever of ≥38°C, and cough, with illness onset in the last 10 days. However, in Ghana, as elsewhere, antipyretics are widely available over‐the‐counter, and self‐medication is common. Therefore, influenza cases that would have presented with ILI may present instead without a fever and not meet the standard ILI or SARI case definition.^11^, ^12^ The resultant missing influenza cases with no fever because of antipyretics may result in an underestimate of the burden of ILI and influenza‐associated ILI.

Our study aimed to assess the frequency of influenza virus detection in persons presenting to a health care facility with the standard ILI case definition with measured fever at presentation, compared with the frequency of detection in those with subjective fever and investigate the role of antipyretics in reducing the sensitivity of the ILI case definition.

## 2 METHODS

### 2.1 Enrolment

From September 2013 to May 2014, we used an expanded ILI case definition to enrol all patients seeking care at four health facilities (one district hospital and three health centres) located in Shai‐Osudoku and Ningo–Prampram Districts (SONPD) in the Greater Accra Region of Ghana. The expanded case definition included patients meeting the ILI case definition (with measured fever >38°C and cough with onset <10 days) but also those who presented with ILI, but with only a history of fever (either previously measured or subjective feverishness) related to the illness and a temperature below 38°C at presentation.

### 2.2 Data collection

After consenting to participate in the study, participants or their legal guardians were interviewed to capture clinical and demographic information using a structured questionnaire administered by trained field staff. Clinical information was also captured from participants' medical records at the health facility and included data on antipyretic use. Patients were asked to indicate the name of any medication they took 24 h prior to visiting the health facility. Patients who were not able to provide the medication name were asked to provide bottle or box.

Questionnaire data were then entered onto Personal Digital Assistants (PDAs), backed up weekly on a Secured Digital Memory Card (SD card) weekly and synchronized with a central database at Noguchi Memorial Institute for Medical Research (NMIMR).

### 2.3 Sample collection, transportation, and laboratory analysis

Nasopharyngeal and oropharyngeal (NP/OP) swabs were collected from each enrolled participant, placed together in a single vial of viral transport medium, stored temporarily for no more than 24 h at 4°C, and transferred into liquid nitrogen at the end of each day. The samples were transported to the National Influenza Centre at NMIMR, Accra, Ghana, for molecular analysis. Ribonucleic acid (RNA) was extracted from samples using QIAamp® Viral RNA Mini Kit commercially available from QIAGEN (Valencia, California, USA) as per manufacturer's instructions and stored temporarily for up to 48 h at 4°C before being tested for influenza viruses by real time reverse‐transcription polymerase chain reaction (rRT‐PCR). Samples were initially tested for influenza A and B viruses as per the CDC protocol for influenza diagnosis.^13^ All influenza A positive samples were then subtyped.

### 2.4 Statistical analysis

We categorized the study participants into three groups: (a) Measured fever ≥38°C or subjective fever and cough with onset within the last 10 days; (b) measured fever ≥38°C and cough with onset within the last 10 days; and (c) measured fever ≥38°C or subjective fever who took antipyretics in the previous 24 h and cough with onset within the last 10 days.

We then calculated the number for each case definition and the proportion which tested positive for influenza and compared the proportions. We calculated the sensitivity, specificity and accuracy of the case definitions.^14^ We further performed chi‐square tests at 95% confidence intervals (CI). A *P* value less than 5% was considered significant. We stored and managed data using a Structured Query Language database (Version 2008, Microsoft, Redmond, WA, USA). We used Statistical Package for the Social Sciences for data analyses (SPSS version 16; IBM Corporation, Armonk, NY, USA).

### 2.5 Ethics approval

The scientific and technical committee and the institutional review board of NMIMR reviewed and approved the research proposal (IRB 00001276) and CDC determined that the surveillance system was not human subjects research (NRD # 2013 6261).

The risks and benefits of participating in the study were discussed with participants prior to enrolment. Informed consent was obtained from all participants or their guardians (for participants aged <18 years). Children aged 5 to 17 years were asked to provide assent. Parent/legal guardian consent alone was obtained for children less than 5 years of age.

## 3 RESULTS

We enrolled 321 participants aged 1 month to 76 years (median = 3 years: Inter Quartile Range [IQR] = 1–11 years), and 51% were female.

### 3.1 Influenza virus detection

Of 321 NP/OP samples processed, influenza viruses were detected in 69 (21%) samples, of which 14 (20%) were influenza A (H1N1) pdm09, two (3%) were influenza A (H3N2), and 53 (77%) were influenza B viruses. There was no statistically significant difference in proportion positive by gender (*P* = .46). Children aged less than 5 years had a lower proportion of samples testing positive for influenza virus (31/187 [16.6%]) compared with participants aged 5 years and older 38/134 [28.4%] (*P* = .007). Participants aged less than 2 years had a lower proportion of samples positive for influenza viruses with nine (10.5%) of 86 compared with 22 (21.8%) of 101 among participants aged 2–4 years (*P* = .04) (Table 1).

**TABLE 1:** Influenza virus detection by age group, Shai‐Osudoku and Ningo Prampram districts, September 2013–may 2014

Variable	Age in years	Number tested	Influenza positive	
			No.	%
	0–1	86	9	10.5
Age groups	2 to 4	101	22	21.8
	5 to 14	63	24	38.1
	15 to 24	23	4	17.4
	25 to 34	25	6	24.0
	35 to 44	15	3	20.0
	45 to 54	3	1	33.3
	55 to 64	4	0	0.0
	≥65	1	0	0.0
	All ages	321	69	21.5%

### 3.2 Accuracy of case definitions for influenza virus detection

Table 2 shows a summary of the accuracy of case definitions for influenza virus detection in Ghana. Of the 85 participants who met the WHO case definition of only measured fever at presentation and cough, 30 (35%) were positive for influenza virus infection. Of the 321 participants, with subjective or measured fever and a cough, 69 (21%) were positive for influenza with an increase of more than twofold compared with the WHO case definition. Of the 321 with the expanded case definition, 142 (44%) visited a health care provider for their illness. Among those who visited a health care provider prior to their visit to the health facility, 110 (34%) visited the pharmacy. A total of 166 (52%) participants took some medication for their illness before visiting the health facility. Among those, antipyretics were the highest recorded with 119 (72%) followed by cough syrup with 32 (19%). Among 236 cases of subjective fever, 77 (33%) took antipyretics within the last 8 h and 159 did not. Of those with subjective fever who took antipyretics within the last 8 h, 16 (21%) were positive for influenza, compared with 23 (14%) of those with subjective fever who did not take antipyretics.

**TABLE 2:** Accuracy of case definitions for influenza virus detection, Shai‐Osudoku and Ningo Prampram Districts, September 2013 to May 2014

Case definition	Number tested	Influenza positive	PPV
			% (CI)
Measured fever ≥38°C or subjective fever and cough	321	69	21.5 (17.35, 26.31)
Measured fever ≥38°C and cough	85	30	35.29 (25.97, 45.89)
Measured fever ≥38°C or subjective fever, and took antipyretics and cough	162	46	28.40 (22.01, 35.78)

*Note*: Subjective fever: Reported fever or feverishness by participant or guardian prior to visiting the facility but has a temperature <38°C at the time the patient got to the facility. Measured fever: Axillary temperature ≥38°C measured by a clinician with a thermometer at the time the patient reported at the facility for the current illness. PPV: positive predictive value.^14^

## 4 DISCUSSION

This study aimed to see if history of antipyretic use can improve the performance of case definitions for influenza. We found, as expected, that a case definition of measured temperature at presentation had the highest specificity and positive predictive value but missed more than half of all influenza positive cases. The addition of subjective fever to the standard ILI case definition added approximately another 57% of influenza cases but also required testing of approximately four times as many patients. However, because those with influenza infections were more likely to take antipyretics in these settings, the positivity rate of those with subjective fever who took antipyretics was higher than among those who did not (21% versus 14%). In this setting, adding subjective fever with antipyretics would add approximately 23% of influenza cases, would require less than a doubling of testing, and could be a good strategy to include if the aim of surveillance is better disease burden determination.

A study conducted in a rural community in North India showed a higher proportion of influenza positivity among ILI patients with a measured temperature >38°C compared with those without fever on presentation.^15^ A stepwise logistic regression analysis of predictors of influenza infection by Monto et al also showed that patients with measured temperature ≥38°C were more than three times likely to test positive for influenza viruses^7^ compared with patients who presented with measured temperature <38°C, but antipyretic use was not reported. In another study by Babcock et al,^16^ subjective fever was found to be one of the common symptoms among patients with influenza, which supports the findings of this study. Self‐medication is a common practice in Ghana and elsewhere, which may increase the use of antipyretics prior to visiting a health care provider.^17^ Although fever is a natural immunological response to an infection, it is usually seen as a sign of worsening diseases condition by most individuals and as such, they take antipyretics to relieve their fears.^18^, ^19^, ^20^, ^21^, ^22^ This might have contributed to antipyretics being the most frequent medication used by participants. Other contributing factors could be the cost of other medication and tight control by request for prescription. In a study conducted in Japan to assess to the detection of influenza cases using fever as a proxy at the airport showed that, 55.6% of confirmed Influenza A(H1N1)pdm09 cases would have been missed because they took antipyretics prior to their arrival.^23^ This is similar to our findings where 23% of influenza cases had taken antipyretics and so did not have fever at the time of screening. In another study that assessed influenza case definitions also concluded that a combination of measured and history or reported fever gave a reasonable balance in terms of sensitivity and specificity of Influenza case definitions.^24^ The findings of their study conform to what we found in our study.

Our study had limitations. First, the assessment of subjective fever was based on the responses given by the participants. Second, our small sample size prevented us from examining other factors associated with antipyretic use, performance of the different case definitions by age groups, and the effect of antipyretics on relative proportions of subtypes, and of fully testing for statistical significance. Third, the prevalence findings are not for a complete year and may not be representative of the rest of the year although that is not the focus of the paper. Finally, we did not include patients with respiratory symptoms who did not have subjective fever or measured fever as this may influence our calculation of sensitivity and specificity.

The burden of influenza in Africa has been increasingly measured and recognized,^25^ highlighting the need to have a sustainable cost‐effective surveillance system. Depending on the goals of the surveillance system, different case definitions can be applied. If the purpose is disease burden rather than strain detection, surveillance can be short‐term and could include those with no fever but recent antipyretic use. More work needs to be done on timing of use of antipyretics and asses if it differentially affects influenza positivity between ages and but also differentially affects one subtype over another.

## AUTHOR CONTRIBUTIONS

**Joseph Asamoah Frimpong:** Conceptualization; data curation; formal analysis; investigation; methodology; writing‐original draft; writing‐review and editing. **William Kwabena Ampofo:** Conceptualization; supervision; writing‐review and editing. **Kofi Mensah Nyarko:** Conceptualization; supervision; writing‐review and editing. **Jazmin Duque:** Validation; writing‐review and editing. **James Aboagye:** Data curation; formal analysis; writing‐original draft. **Kwadwo Koram:** Supervision; writing‐review and editing. **Marc‐Alain Widdowson:** Supervision; validation; writing‐review and editing.

## CONFLICT OF INTEREST

We also wish to confirm that there are no known conflicts of interest associated with this publication and there has been no significant financial support for this work that could have influenced its outcome.

### PEER REVIEW

The peer review history for this article is available at https://publons.com/publon/10.1111/irv.13068.

## Data Availability

The data that support the findings of this study are available from the corresponding author upon reasonable request.
